# The fertility willingness and acceptability of preimplantation genetic testing in Chinese patients with autosomal dominant polycystic kidney disease

**DOI:** 10.1186/s12882-020-01785-x

**Published:** 2020-04-25

**Authors:** Mingji Sun, Cheng Xue, Yunhui Lu, Yiyi Ma, Ting Pan, Xiaoliu Wang, Li Fan, Jiandong Shen, Yan Hao, Danxia Zheng, Junhua Li, Mingxu Li, Yaping He, Changlin Mei

**Affiliations:** 1 Department of Nephrology, Changzheng Hospital, Second Military Medical University, 415 Fengyang Road, Shanghai, 200003 China; 2 Department of Nephrology, The First Affiliated Hospital, Sun Yat-sen University, NHC Key Laboratory of Nephrology (Sun Yat-sen University), Guangdong Provincial Key Laboratory of Nephrology, Guangzhou, 510080 China; 3 The Center of Reproductive Medicine, the First Affiliated Hospital of Nanjing Medical University, State Key Laboratory of Reproductive Medicine, 16 Yongqing Lane, Nanjing, 210029 China; 4 Reproductive Medicine Center, Department of Obstetrics and Gynecology, the First Affiliated Hospital of Anhui Medical University, 218 Jixi Road, Hefei, 230022 China; 5 Division of nephrology, Peking University Third Hospital, 49 Huayuanbei Road, Beijing, 100191 China; 6 Division of Nephrology, Tongji hospital, Tongji Medical College, Huazhong University of Science and Technology, 1095 Jiefang Road, Wuhan, 430030 China; 7 Division of Nephrology, Sixth medical center of general hospital PLA, 6 Fucheng Road, Beijing, 100048 China; 8 School of Public Health, Shanghai Jiao Tong University, 227 South Chongqing Road, Shanghai, 200025 China

**Keywords:** Autosomal dominant polycystic kidney disease, Genetics, Life quality, Preimplantation genetic testing

## Abstract

**Background:**

With the development and progression of genetic technology, preimplantation genetic testing (PGT) has made it possible to block the inheritance of autosomal dominant polycystic kidney disease (ADPKD) as early as possible. However, we need to know the patients’ fertility intentions and their acceptance of PGT.

**Methods:**

A questionnaire survey was conducted to collect data on the basic demographic data, quality of life, social support, fertility willingness, and level of understanding of genetic testing for blocking the inheritance of ADPKD among patients aged 18–45 years in seven hospitals from January 2018 to December 2018. After verification, statistics were calculated.

**Results:**

A total of 260 patients with ADPKD were interviewed, including 137males (52.7%) and 123 females (47.3%). The overall fertility willingness rate was low (*n* = 117, 45.0%). The proportion of married patients aged 25–34 years that were at the optimal reproductive age but did not yet have children was relatively high (*n* = 77, 67.0%). The fertility intentions of ADPKD patients were significantly influenced by age (OR: 0.101, 95% CI 0.045–0.225, *P <* 0.001) and education level (OR: 2.134, 95% CI 1.162–3.917, *P =* 0.014). Among patients who are willing to have children, 207 (79.6%) of them would choose PGT technology. Among those who were not sure whether they would choose PGT technology, the first major concern was technical safety (49.2%).

**Conclusions:**

The reproductive desire of childbearing ADPKD patients in China was low. Strengthening the health education of ADPKD genetic knowledge and reducing the cost of related technologies may improve the fertility intentions and reduce the barriers to acceptance of PGT.

## Background

Autosomal dominant polycystic kidney disease (ADPKD) is the most common hereditary kidney disease in humans. It occurs in all races with a prevalence estimated to be between 1:400 and 1:1000 [1, 2]. ADPKD is the fourth leading cause of end-stage renal disease (ESRD) in China [3]. Its incidence is 10 times that of sickle cell anemia, 15 times that of cystic fibrosis, and 20 times that of Huntington’s disease [4]. It is also more common than Down syndrome, hemophilia and myotonic dystrophy [5]. More than 80% of cases are caused by mutations in the patients’ parents’ disease-causing genes [6]. The early clinical manifestations of ADPKD are possible under 30 years of age and include hypertension, gross hematuria, urinary tract infection and other symptoms [7]. By the age of 65, nearly 45 to 70% of patients will develop ESRD [8]. There are currently few effective drugs to treat the disease, and the treatment in the early stages of ADPKD mainly includes blood pressure control and regular follow-up [5, 9]. However, research on the physiological and psychological burden of patients in the early stages of ADPKD is very limited, and the conclusions are conflicting [10]. Obviously, in the early stages of the disease, as a social individual, it is a critical period for ADPKD patients to set up families and have children. Some studies have shown that even in the early stages, polycystic kidney disease (PKD) imposes a physical and psychological burden on patients, resulting in a decline in their quality of life. If doctors are not aware of this, patients may feel frustrated [10–12]. Patients’ fear of the disease and feelings of helplessness, as well as doctors’ insufficient health education on ADPKD and its relevant knowledge, may both affect the fertility willingness of ADPKD patients because of their fear of the inheritance of the disease and the complications of pregnancy and even hinder them from forming a family [13–15]. Therefore, it is of great significance to understand the fertility intentions of ADPKD patients and to analyze the factors affecting their fertility to improve their quality of life.

On the other hand, assisted reproductive technology, represented by the third generation of in vitro fertilization (IVF) and preimplantation genetic testing (PGT), perfectly combines the development of genetic technology with clinical disease intervention, which makes it possible for patients who have fertility issues to have a child, and the age of childbearing is gradually increasing. China’s relevant laws and regulations on PGT technology are similar to those of the UK, which recognize the operation of embryos within 14 days and have a qualification certification system for implementing PGD technology institutions. PGT brings great benefits to ADPKD patients and their families [16–19]. In recent years, we have successfully blocked the inheritance of ADPKD for several couples by using multiple annealing and looping-based amplification cycles (MALBAC)-PGT technology in cooperation with the Department of Obstetrics and Gynecology Reproduction. Several healthy infants have been born with a successful blocking of the inheritance of the ADPKD gene [20, 21]. However, there are few studies on how well people with ADPKD understand these new technologies and whether they are psychologically acceptable or financially affordable.

Considering that different countries have different social security systems, economic development and fertility concepts, there may be obvious differences in fertility intentions [22]. To understand the fertility desire and attitude towards PGT of ADPKD patients of childbearing age in China, we conducted this multicenter cross-sectional study in seven PKD research centers with PGT qualification.

## Methods

### Sample size calculation

Because the main focus of this study is the fertility desire of ADPKD patients in China, the proportion of fertility desire is the main observation index. Considering that there are no previous data to reference the fertility intentions of people with ADPKD of childbearing age, the estimated overall rate, π, is 50%, and the allowable error, δ, is 15% (π = 0.075, α = 0.05, Ζα/2 = 1.96). According to the sample content estimation formula, when estimating the population rate, *n*= $\left(\frac{Z\alpha /2}{\delta}\right)2$.π(1-π), the estimated sample size is 171.

### Inclusion and exclusion criteria

Seven centers identified the respondents according to the same criteria for inclusion and exclusion. The selection criteria were as follows: (1) patients with an age between 18 and 45 years for whom gender and glomerular filtration rate information was available; (2) patients with or without a family history of ADPKD and with ADPKD diagnosed clinically by ultrasonography, computed tomography (CT) and magnetic resonance imaging (MRI), or genetic detection [6]; and (3) patients who were willing to sign the informed consent. The exclusion criteria were as follows: (1) patients with severe physical and mental disorders affecting cognitive function, (2) patients with visual acuity and hearing impairment who could not complete the survey, and (3) patients who refused to sign the informed consent form.

### Study design and setting

From January 2018 to December 2018, patients with ADPKD were admitted to Shanghai Changzheng Hospital, the First Affiliated Hospital of Sun Yat-sen University, the Sixth Medical Center of the General Hospital of the Chinese People’s Liberation Army, the Tongji Hospital of Tongji Medical College of Huazhong University of Science and Technology, Jiangsu People’s Hospital, the Third Hospital of Peking University and the First Affiliated Hospital of Anhui Medical University. Patients were interviewed by face-to-face questionnaires. These seven hospitals cover six different provinces and cities in China and are clinical centers involved in Using Preimplantation Genetic Diagnosis in ADPKD Patients: a Multicenter Clinical Trial (ESPERANCE) (NCT02948179). The research project and questionnaire were approved by the Ethics Committee of Shanghai Changzheng Hospital. Before participating in the questionnaire, patients had to read the informed consent form, fully understand all the contents, make sure they were willing to participate in the study, and know that they could choose not to participate in this study and that any medical treatment and welfare would not be affected.

After completing routine clinical diagnosis and treatment for patients with ADPKD, nephrologists informed the patients of the questionnaire survey and asked the patients whether they agreed to participate. If the patients agreed, they were directed to the investigators, signed the informed consent form and entered the questionnaire survey stage. If the patient refused, the doctor would ask the reason for the refusal, but would not force the patient to answer. Before filling in the questionnaire, the investigators would make a basic introduction of PKD and the process of PGT and prenatal diagnosis (PND) technology, and confirmed that the patients had a basic and accurate understanding of relevant concepts. During the questionnaire survey, patients could also raise any questions about related concepts at any time.

### Questionnaire

After consulting the literature and experts, the researcher designed a “Questionnaire on fertility intention of patients with polycystic kidney disease and cognition of preimplantation gene detection technology”, which could investigate the sociodemographic data, quality of life and social support of patients with ADPKD. The questionnaire is provided in the “Additional file 1”.

The first part of the questionnaire was personal information collection. There were 10 items in total, including gender, marital status, age of first marriage, the birth date of the respondents and their spouses (if any), education level, agricultural and non-agricultural household type, household registration location, occupation, whether they were the only child, and family annual income.

The second part of the questionnaire was the Autosomal Dominant Polycystic Kidney Disease–Impact Scale (ADPKD-IS™). The scale, developed by Dorothee Oberdhan (2017) et al., is suitable for a comprehensive assessment of the Health-related quality of life (HRQoL) and overall disease burden of patients with ADPKD. There are three dimensions: the physical domain, the emotional domain,and the fatigue domain. Each dimension was scored 1–5 points from light to heavy according to the degree of disease impact. The test-retest reliability coefficient of the scale was above 0.86, and the consistency of all dimensions and the whole was above 0.85 [13].

The third part of the questionnaire was the Social Support Revalued Scale (SSRS). It was developed by Xiao and Yang in 1986 and used to measure social support. The SSRS comprises 10 items divided into 3 subscales: objective support subjective support, and use of social support. Reliability is sound with a Cronbach’s α of 0.888 and a test-retest reliability coefficient of 0.92 [23]. This instrument has been widely used by Chinese researchers [24, 25]. The cumulative score of the SSRS ranges from 11 to 66 points; higher scores indicate greater social support.

The last four parts of the questionnaire were designed on the basis of the questionnaire carried out by Swift [26] in the U.K. and combined with China’s national conditions. They were mainly related to the understanding and acceptance of PGT technology and medical insurance costs.

### Statistical analyses

Epi Data 3.1 was used to manage the data. The statistical software SPSS 24.0 (IBM) was used for statistical analysis and processing. The age, glomerular filtration rate and social support scores of subjects with normal distribution were measured by mean (±standard deviation) descriptively. The scores on ADPKD-IS™ dimensions that did not conform to the normal distribution are expressed as the median (Q1, Q3). Student’s t-test, chi-squared tests, and the Mann-Whitney U test were used to compare the differences between the groups with and without fertility intentions. Enumeration data and the proportion of different groups were described and counted by the number and percentage. The 12 factors that may affect the fertility intentions of patients were analyzed by single-factor and multivariable logistic regression analysis. *P* < 0.05 indicated a statistically significant difference.

## Results

### Characteristics of participants

#### Basic information of the respondents

In this study, a total of 400 questionnaires were distributed, and 326 questionnaires were recovered by the convenience sampling method, with a recovery rate of 81.5%. After screening, there were 260 valid questionnaires, with an effective rate of 65.0%. Among the 66 invalid questionnaires, 65 were excluded because their age did not meet the inclusion criteria, and one was excluded because more than 10% of their items were missing. During the implementation of the questionnaire, 13 patients refused the invitation to participate. Reasons included lack of time and interest.

**Fig. 1:**
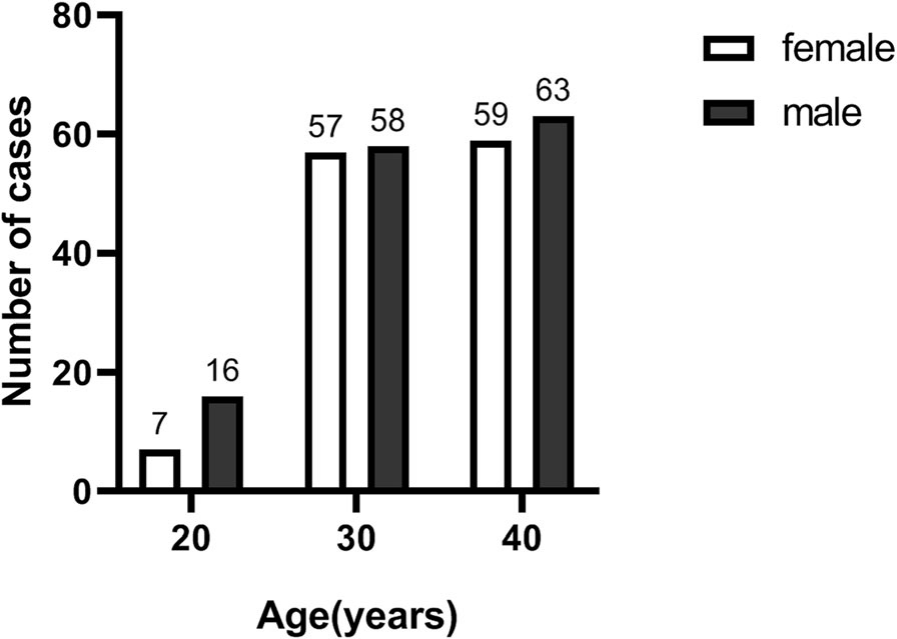
The age distribution of the respondents. Among them, 137 were males and 123 were females. “20” represents the age range of 18~24 years, “30” represents the age range of 25~34 years, and “40” represents the age range of 35~45 years

A total of 260 patients with ADPKD were investigated, including 137 males (52.7%) and 123 females (47.3%). The age distribution ranged from 20 to 45 years, with an average of 33.9 ± 6.6 years, as shown in Fig. 1. Among the respondents, the highest proportion of education is junior college or undergraduate, accounting for 58.5%. The number of registered patients with creatinine in the last month was 251. The average eGFR obtained by the CKD-EPI formula was 96.4 ± 48.4 ml/min/1.73m^2^. Other basic information is shown in Table 1.

**Table 1:** Demographics of the respondents [n (%)]

Parameter	n (%)
Gender	
Female	123 (47.3)
Male	137 (52.7)
Age (y)	
18~24	23 (8.8)
25~34	115 (44.2)
35~45	122 (46.9)
CKD stage	
G1	144 (55.4)
G2	44 (16.9)
G3a	16 (6.2)
G3b	18 (6.9)
G4	17 (6.5)
G5	12 (4.6)
Unknown	9 (3.5)
Degree of education	
Junior high school and below	45 (17.3)
High school	30 (11.5)
College and undergraduate	152 (58.5)
Postgraduate and above	31 (11.9)
Unknown	2 (0.8)
The nature of household registration	
Non-urban	77 (29.6)
Urban	183 (70.4)
They are only child or not	
Yes	114 (43.8)
No	146 (56.2)
ADPKD family history	
Yes	198 (76.2)
No	41 (15.8)
Uncertain	21 (8.1)

#### Marriage and childbearing

Among the respondents, 65 (25.0%) patients were unmarried, 189 (72.7%) were in their first marriage, and 6 (2.3%) were remarried, divorced or widowed. A total of 143 (55.4%) patients had children, and the average number of children was 1.3 ± 0.6; 117 (45.0%) patients had no children. A total of 101 (38.8%) patients had one child, and 42 (16.2%) patients had two or more children. Only 49 of these children had been tested for PKD, 28 were negative, 17 were positive, 3 were suspicious, and 114 had not. Marriage and childbearing in different age groups are shown in Tables 2 and 3.

**Table 2:** Marital status at different ages [n (%)]

Age (years)	Marital status		Total
	Unmarried (%)	Married (%)	
18~24	22(95.7)	1(4.3)	23
25~34	33(28.9)	81(71.1)	114
35~45	10(8.2)	112(91.8)	112
Total	65(25.1)	194(74.9)	259

**Table 3:** Fertility in different ages [n (%)]

Age (years)	Grouping by number of children			Total
	Childless (%)	One child (%)	Two or more children (%)	
18~24	23(100.0)	0(0.0)	0(0.0)	23
25~34	77(67.0)	30(26.1)	8(7.0)	115
35~45	17(14.7)	71(59.5)	34(25.9)	122
Total	117(45.0)	101(38.8)	42(16.2)	260

#### HRQoL of patients

**Fig. 2:**
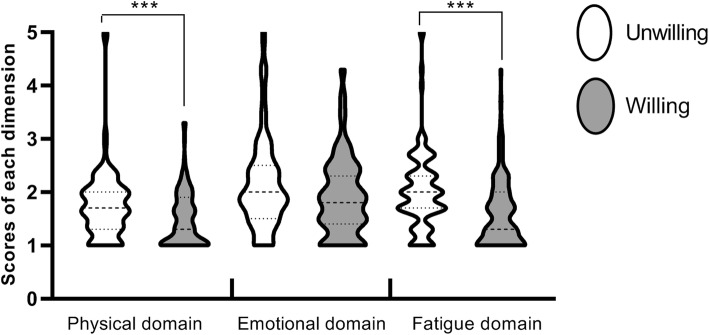
Scores of each dimension in the group with or without fertility intentions. The health-related quality of life (HRQoL) of the patients was assessed by ADPKD-ISTM. Each domain score can range from 1 to 5. Depending on the burden of the disease on the patient, a score of 1 indicates not difficult at all or not bothered at all, and a score of 5 indicates extremely difficulty or extremely bothered. The scores are presented as the median (min to max) (***P < 0.001)

Using the ADPKD-IS™, the HRQoL status of patients with ADPKD was investigated. The average scores of the physical domain, emotional domain and fatigue domain were 1.6 ± 0.6, 2.0 ± 0.8 and 1.8 ± 0.7, respectively. Detailed results are shown in Fig. 2.

#### Social support of patients

By using SSRS, the total score of social support for ADPKD patients of childbearing age was 38.0 ± 7.9. The average score of the objective support dimension was 9.0 ± 3.1, the subjective support dimension score was 22.0 ± 5.0, and the average score of the social support utilization dimension was 7.0 ± 2.0.

#### Fertility willingness

**Fig. 3:**
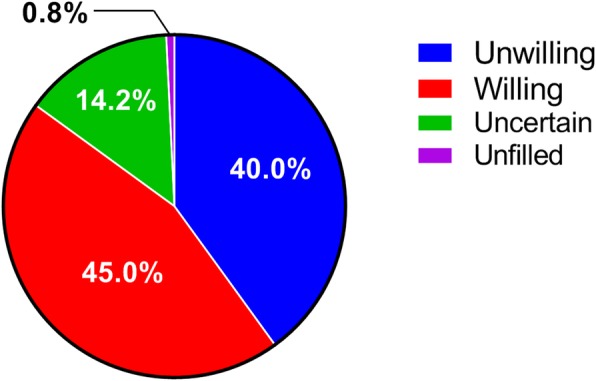
Fertility willingness of childbearing-age ADPKD patients (*n* = 260)

Designed 0–10 scale questions were used to understand the intensity of the fertility willingness of patients. A score of 0 represented total disinclination, and a score of 10 represented great desire. ADPKD patients had an average score of 5.0 ± 3.9. The fertility intentions of ADPKD patients of childbearing age are shown in Fig. 3.

**Fig. 4:**
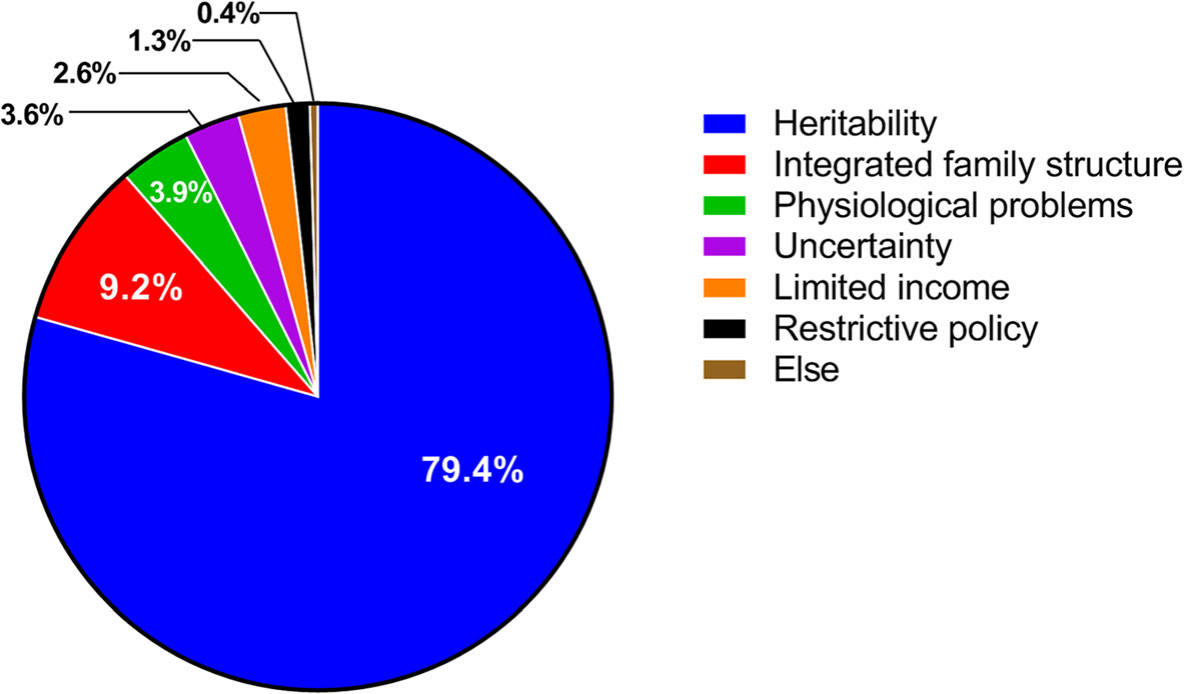
Reasons for patients’ self-reported lack of fertility desire

The main reasons for the self-reported lack of fertility willingness were the worry about the inheritance of PKD (79.4%), integrated family structure (9.2%)and physiological problems (including old age, combined with other diseases) (3.9%). (Fig. 4).

Of the patients that planned to have a child in the future, 248 (96.1%) were worried that the child would also have PKD, 2 (0.8%) were not worried, and 10 (3.8%) were uncertain. The average score was 9.1 ± 2.0 for the willingness to block the PKD gene in the next generation through the existing technical means (0–10 points on a scale from low to high).

#### Cognition and acceptance of PGT

Before the questionnaire survey, 157 (60.4%) of the respondents knew that the inheritance of the PKD gene could be blocked by PGT technology, 92 (35.4%) did not know, and 11 (4.2%) expressed uncertainty. After introduction to the basic processes of PGT, 138 (53.1%) patients were not clear about the safety of the technology, 65 (25.0%) patients felt fundamentally safe, 29 (11.2%) patients felt less dangerous, and 23 (8.8%) patients felt very safe.

If future births were to occur, 207 (79.6%) patients said they would choose PGT technology, 8 (3.1%) patients said they would not, and 44 (16.9%) patients said they were uncertain. Among those who chose PGT technology, 183(88.0%) patients were willing to pay approximately ¥50,000 for PGT and IVF, 20 (9.6%) were uncertain and 5 (2.4%) were unwilling. Among those who were not sure whether they would choose PGT technology, their main concerns were technical safety (49.2%), cost (22.0%) and the technical accuracy (13.6%).

#### The change in fertility willingness if PGT would be included into medical insurance

If PND and PGT can be included in national medical insurance reimbursement, 153(58.8%) of the patients would have a(nother) baby and choose PGT at the same time, 63 (24.2%) of the patients would not have a(nother) children, and 31 (11.9%) of the patients would have a(nother) baby and choose PND at the same time.

### Analysis of the influencing factors of fertility intentions

#### Univariate analysis of influencing fertility willingness

One-way chi-squared analysis was conducted on 12 factors that might affect fertility intentions, including gender, age, CKD stage, education level, marital status, annual family income, the nature of household registration, whether the patients were only children, the patients’ health-related quality of life, social support, and the awareness of PGT and PND. Age, CKD stage, education level, marital status, whether they were an only child, the patients’ health-related quality of life, social support, and PGT and PND awareness were statistically significant between the fertility groups (*P* values all < 0.05). The specific results are shown in Table 4.

**Table 4:** Univariate analysis of influencing fertility willingness

Variable	Effective sample size	χ^2^	*P value*
Gender	258	3.740	0.154
Age	258	94.655	<0.001
CKD stage	249	25.139	0.005
Degree of education	256	22.495	0.001
Marital status	257	39.144	<0.001
Annual family income	255	3.563	0.736
The nature of household registration	258	0.168	0.919
Only child or not	258	8.425	0.015
Health-related quality of life	258	13.238	0.001
Social support	258	16.328	<0.001
Cognitive status of PGT	258	6.163	0.046
Cognitive status of PND	258	9.551	0.008

#### Multivariate analysis of influencing fertility willingness

**Fig. 5:**
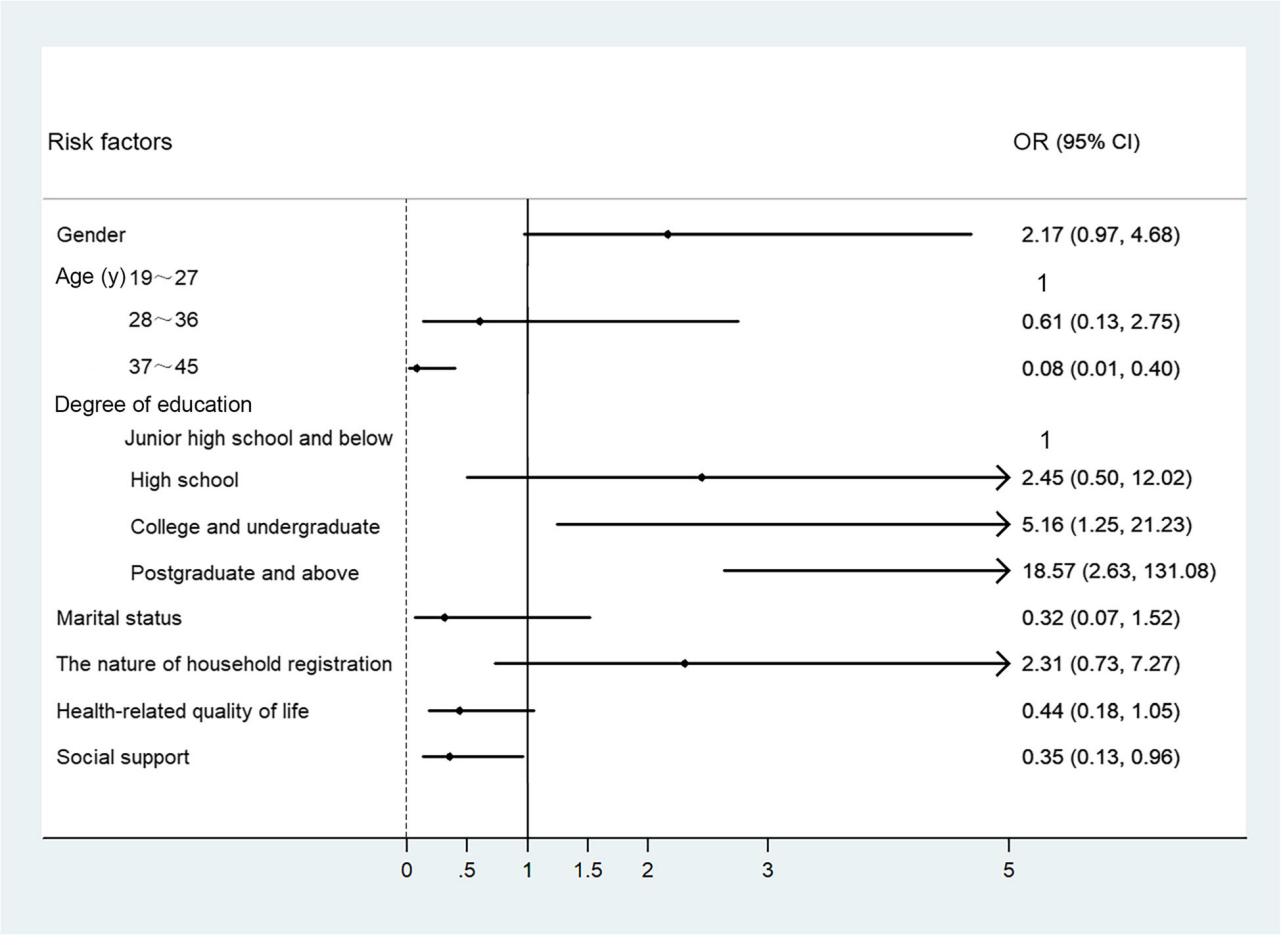
Binary Logistic regression analysis of influencing fertility willingness. The 19~27 years group is the baseline for age comparisons (P<0.001), 28~36 years group (*P* = 0.515), 37~45 years group (*P* = 0.002). Junior high school and below is the baseline for comparisons of degree of education (*P* = 0.029), High school (*P* = 0.271), College and undergraduate (*P* = 0.023), Postgraduate and above (*P* = 0.003). Gender (*P* = 0.059). Marital status (*P* = 0.150). The nature of household registration (*P* = 0.154). Health-related quality of life (*P* = 0.065). Social support (*P* = 0.062).Internal assignment(No desire to have children =0, Willingness to bear =1)

Multiple factors affecting fertility intentions were analyzed by binary logistic regression. Twelve factors, including gender, age, CKD stage, educational level, marital status, annual family income, the nature of household registration, whether they were only children, the patients’ health-related quality of life, social support, and the awareness of PGT and PND, were included in the binary logistic regression model. The results showed that the fertility intentions of ADPKD patients were significantly influenced by age group (OR: 0.186, 95% CI 0.091–0.381, *P <* 0.001) and education level (OR: 2.438, 95% CI 1.355–4.385, *P =* 0.003). The older the patients were, the lower their fertility desire was, and the higher their educational level was, the more likely they were to have a desire for fertility. The results of the stratified analysis of different age groups and educational levels are shown in Fig. 5.

## Discussion

### Fertility willingness

Childbearing and reproduction are basic needs of human beings. For most Chinese families, childbearing is one of the most important functions of marriage and family. Considering the existing research, whether there is the influence of disease or not, people’s fertility intention is strongly related to age, and questionnaire is mainly designed to understand the fertility intention of ADPKD patients and the cognition of PGT technology, so the control study with healthy people was not included in the study design [27, 28]. This study showed that only 45.0% (117/260) of ADPKD patients at reproductive age had fertility intentions. This rate was significantly higher than the 17% of 58 UK ADPKD patients at CKD stages 1–4, with an average age of 44.6 ± 12.7 years in Swift et al.’s study [26]. However, it was lower than that of 51% of people with neurofibromatosis type 1 in Ponder M et al.’s study [29]. It was also lower than that in Kraus EM et al.’s study of 35 mothers of children with hemophilia, which showed that 57% of the mothers had family planning intentions [30] The intensity of the fertility desire of 5.0 ± 3.9 points also indicates that the fertility desire of ADPKD patients in China is low.

Patients’ self-reported primary reasons for not wanting to have children were genetic worries about PKD. The percentage of patients with reproductive desires increased from 45.0 to 71.5% after receiving information on the basic processes and action of PGT and PND technologies. Therefore, the information on assisted reproductive technologies such as PGT and PND can reduced the patient’s concerns and significantly improved the fertility intentions of patients with ADPKD during pre-pregnancy counseling.

Both univariate analysis and multifactor logistic regression analysis showed that both age and education level were directly associated with the fertility intentions of ADPKD patients. Understandably, the combination of psychological factors such as age, a decline in renal function and hypothalamus-pituitary-gonadal axis function, depression, and a decline in sexual function will reduce fertility willingness and the possibility of fertility [31, 32]. However, in this study, we found that although the proportion of married patients aged 25–34 years at the optimal reproductive age was relatively high (71.1%), the proportion without children was also relatively high (67.0%); that is to say, most of them were married but did not yet have children. Therefore, it is necessary to give proper prenatal guidance and counseling to the patients of this age group to avoid missing the best childbearing period.

### Understanding of and attitude towards PGT and PND technology

Before the questionnaire survey, the proportion of people who knew about PGT and PND technology was approximately 60%. About 40% of the PKD patients of childbearing age did not know how to block the inheritance of the PKD gene in the next generation. Therefore, it is necessary to increase the education in and publicity of PGT-related knowledge in the PKD population. 79.6% of PKD patients would choose PGT if they had children, which is significantly higher than the percentage in Swift’s study of 96 UK patients with PKD, which showed that 50–63% would choose PGT [26]. This indicated that the new technology of blocking the genetic inheritance of PKD had high acceptance and need in PKD patients in China. However, we found that the proportion of people who were not clear about the safety of PGT was still more than 50% after introducing the basic processes of PGT, and the main concern of those patients who were not sure whether they would choose PGT was the safety of PGT. In addition to worrying about technical safety, cost was the second largest concern of patients because nearly half of the 260 PKD patients surveyed received annual incomes of less than ¥100,000, and the cost of PGT and a cycle of IVF of approximately ¥50,000 was obviously a large financial burden for them.

### Limitations

Because this study adopts the method of convenient sampling to carry out questionnaire survey, it is unable to carry out the survey on all patients during the study period, so there is a certain selection bias. Among the respondents, 27.0% (70 / 260) had been tested for PKD gene, but many of them did not carry relevant test reports. Therefore, this study did not grasp the gene mutation of the participants, so it was not clear whether their fertility intention was related to different gene mutation types. Before we make a basic introduction of PGT and PND technology, we asked whether the respondents knew that such technology could block ADPKD inheritance before, but we did not test on their baseline knowledge regarding PGD and PND.

## Conclusions

Due to the fear of inheritance of ADPKD in the next generation and insufficient knowledge of gene blocking techniques, the proportion of fertility or reproductive desire and the intensity of the fertility desire of ADPKD patients of childbearing age in China are at a low level. Improving the quality of life of patients, increasing the publicity of knowledge related to PKD genetic testing, and reducing the economic burden of related technologies may effectively improve the fertility intentions of patients and reduce the barriers to the acceptance of PGT technology. Particular attention should be paid to patients aged 25–34 years who are at the optimal reproductive age so as not to miss the best childbearing period.

## Supplementary information

**Additional file 1.** Questionnaire on fertility intention of patients with polycystic kidney disease and cognition of pre-implantation genetic testing technology. The questionnaire includes the basic demographic data, the Autosomal Dominant Polycystic Kidney Disease–Impact Scale (ADPKD-IS™), the Social Support Revalued Scale (SSRS), fertility willingness, and level of understanding of genetic testing for blocking the inheritance of ADPKD.

## Data Availability

The data that support the findings of the current study (i.e. the completed questionnaires) is not publicly available due to the Institutional Review Board restrictions. Data are however available from the authors upon reasonable request and with permission of the Institutional Review Board.

## Abbreviations

PGT: Preimplantation genetic testing
ADPKD: Autosomal dominant polycystic kidney disease
ESRD: End-stage renal disease
PKD: Polycystic kidney disease
IVF: In vitro fertilization
MALBAC: Multiple annealing and looping-based amplification cycles
MRI: Magnetic resonance imaging
PND: Prenatal diagnosis
ADPKD-IS™: Autosomal dominant polycystic kidney disease–impact scale
HRQoL: Health-related quality of life
SSRS: Social support revalued scale
U.K.: United Kingdom
eGFR: estimated Glomerular filtration rate
CKD-EPI: Chronic kidney disease epidemiology collaboration
CKD: Chronic kidney disease
