# Supplementary material for: The fertility willingness and acceptability of preimplantation genetic testing in Chinese patients with autosomal dominant polycystic kidney disease
Source: BMC Nephrol. 2020 Apr 25;21:147. doi: 10.1186/s12882-020-01785-x (PMC7183678; doi:10.1186/s12882-020-01785-x)
Supplement: Supplementary file 1 — Additional file 1. Questionnaire on fertility intention of patients with polycystic kidney disease and cognition of pre-implantation genetic testing technology. The questionnaire includes the basic demographic data, the Autosomal Dominant Polycystic Kidney Disease–Impact Scale (ADPKD-IS™), the Social Support Revalued Scale (SSRS), fertility willingness, and level of understanding of genetic testing for blocking the inheritance of ADPKD. [file 12882_2020_1785_MOESM1_ESM.docx]

**Questionnaire on fertility intention of patients with polycystic kidney disease and cognition of pre-implantation genetic testing technology**

Guidance:

1 There is no right or wrong answer to the questionnaire, just fill it out according to your actual situation. If you have any questions, please consult the staff around you.

2 Your answer is very important for our research work. I hope you can fill it out truthfully.

3 Except for special instructions, this questionnaire is a single-choice topic, which can be typed at □ or number, or filled out according to the requirements of the subject.

4 It's a great honor for us to listen to your thoughts. We solemnly promise that the contents of the questionnaire will be used for research purposes only. All information will be kept confidential and will not disclose any personal identity information. Please fill in the form according to your actual situation. Thank you for your cooperation and support!

**Personal information:**

A01. Your gender：1: male 2: female

A02. Your marital status：1: unmarried（Jump to A04） 2:first marriage 3: remarriage 4: divorce 5: widowed

A03. Your first marriage time： year month

|  | Yourself | Your Spouse(If married) |
| --- | --- | --- |
| A04. Date of birth | year month | year month |
| A05. Educational level | □Below junior high school  □Junior middle school  □High school  □College and undergraduate  □Postgraduate and above | □Below junior high school  □Junior middle school  □High school  □College and undergraduate  □Postgraduate and above |
| A06.The nature of household registration | □Non-urban household registration □Urban residence registration | □Non-urban household registration □Urban residence registration |
| A07. Residential City | city | city |
| A08. Your occupation is (before or at present) | □Worker  □Farmer  □Doctors/Teachers/Technicians  □Administrative Cadres/Civil Servants  □Clerk  □Self-employed labourer/ Engage in trade  □Flexible employment  □Housekeeping/Unemployed personnel/School students  □Other | □Worker  □Farmer  □Doctors/Teachers/Technicians  □Administrative Cadres/Civil Servants  □Clerk  □Self-employed labourer/ Engage in trade  □Flexible employment  □Housekeeping/Unemployed personnel/School students  □Other |
| A09. Only child or not | □Yes □No | □Yes □No |

**Autosomal Dominant Polycystic Kidney Disease-Impact Scale**

**(ADPKD-IS^™^)**

This questionnaire helps to evaluate the impact of Autosomal Dominant Polycystic Kidney Disease (PKD) and its related symptoms on your life.

Please select or mark the one box that best describes your answer. Please be sure to respond to **every** question.

| As it relates to PKD, over the past **two weeks,** how ***difficult*** was it for you to... | Not difficult  at all | A little difficult | Somewhat difficult | Very difficult | Extremely difficult |
| --- | --- | --- | --- | --- | --- |
| *1. …engage in leisure activities and mild exercises such as gardening, walking, or stretching?* | ❑ 1 | ❑ 2 | ❑ 3 | ❑ 4 | ❑ 5 |
| *2. …complete a full day’s work at your job or at home (housework, such as vacuuming or laundry)?* | ❑ 1 | ❑ 2 | ❑ 3 | ❑ 4 | ❑ 5 |
| *3. …conduct daily activities as usual, regardless of pain you experienced due to your PKD?* | ❑ 1 | ❑ 2 | ❑ 3 | ❑ 4 | ❑ 5 |
| *4. …complete everything you wanted to do in a day because you felt tired or exhausted?* | ❑ 1 | ❑ 2 | ❑ 3 | ❑ 4 | ❑ 5 |
| *5. …perform more intense physical activities such as lifting, running, or playing sports?* | ❑ 1 | ❑ 2 | ❑ 3 | ❑ 4 | ❑ 5 |
| *6. …accept PKD as a part of your life?* | ❑ 1 | ❑ 2 | ❑ 3 | ❑ 4 | ❑ 5 |
| *7. …cope with feeling guilty about the impact of your PKD on your children or other family members?* | ❑ 1 | ❑ 2 | ❑ 3 | ❑ 4 | ❑ 5 |
| *8. …sleep because of your PKD?* | ❑ 1 | ❑ 2 | ❑ 3 | ❑ 4 | ❑ 5 |
| As it relates to PKD, over the past **two weeks**, how ***bothered*** were you by... | Not bothered at all | A little bothered | Somewhat bothered | Very bothered | Extremely bothered |
| *9. …the size and/or shape of your abdomen?* | ❑ 1 | ❑ 2 | ❑ 3 | ❑ 4 | ❑ 5 |
| *10. …feeling exhausted or fatigued?* | ❑ 1 | ❑ 2 | ❑ 3 | ❑ 4 | ❑ 5 |
| *11. …feeling anxious by knowing that your PKD may get worse?* | ❑ 1 | ❑ 2 | ❑ 3 | ❑ 4 | ❑ 5 |
| *12. …feeling sad about having PKD?* | ❑ 1 | ❑ 2 | ❑ 3 | ❑ 4 | ❑ 5 |
| *13. …feeling full before your appetite is satisfied?* | ❑ 1 | ❑ 2 | ❑ 3 | ❑ 4 | ❑ 5 |
| *14. …needing to urinate frequently and/or urgently?* | ❑ 1 | ❑ 2 | ❑ 3 | ❑ 4 | ❑ 5 |
| *15. …needing to modify your lifestyle (such as the way you sleep or limiting exercise) because of discomfort or pain from your PKD?* | ❑ 1 | ❑ 2 | ❑ 3 | ❑ 4 | ❑ 5 |
| *16. …your PKD-related pain?* | ❑ 1 | ❑ 2 | ❑ 3 | ❑ 4 | ❑ 5 |
| *17. …feeling tired on your drive to/from school or work or when running errands?* | ❑ 1 | ❑ 2 | ❑ 3 | ❑ 4 | ❑ 5 |
| *18. …feeling fatigued the next day even after a night’s sleep?* | ❑ 1 | ❑ 2 | ❑ 3 | ❑ 4 | ❑ 5 |

**Social Support Revalued Scale（SSRS）**

Guidance: The following questions are used to reflect the support you have received in society. Please fill in the following questions according to the specific requirements and your actual situation. Thank you for your cooperation.

1. How many close friends do you have that you can support and help? (Single option)

(l) None of them.

(2) l-2

(3) 3-5

(4) Six or more.

2. Who do you live with in the past year? (Single option)

(l) Keep away from others and live in a single room.

(2) Residences change frequently, and most of the time they live with strangers.

(3) Living with classmates, colleagues or friends.

(4) Live with your family.

3. You and your neighbors: (Single option)

(l) Never care about each other, just nodding acquaintances.

(2) May be a little concerned about each other's difficulties. (3) Some neighbors are very concerned about you.

(4) Most neighbors care about you.

4.You and your colleagues: (Single option)

(l)Never care about each other, just nodding acquaintances.

(2) May be a little concerned about difficulties.

(3) Some colleagues are very concerned about you.

(4) Most of your colleagues are very concerned about you.

5. Support and care from family members (Put “√”in the appropriate box “√”)

|  | Nothing | Very few | General | Full support |
| --- | --- | --- | --- | --- |
| A. Husband and wife (lover) |  |  |  |  |
| B. Parent |  |  |  |  |
| C.Children |  |  |  |  |
| D. Brothers and sisters |  |  |  |  |
| E. Other members (e.g. sister-in-law) |  |  |  |  |

6. In the past, when you were in a difficult situation, the sources of financial support and help in solving practical problems were as follows:

(l) No source

(2) The following sources: (Multiple options)

A. Spouse;B. Other family members;C. Relative;D. Friend;E. Colleague;F. Work unit;G. Official or semi-official organizations such as Party, League, Trade Union, etc.;H. Non-official organizations such as religious and social organizations;

I. Other (please list)__________________

7. In the past, when you were in a difficult situation, the sources of comfort and concern you had received were:

(l) No source

(2) The following sources: (Multiple options)

A. Spouse;B. Other family members;C. Relative;D. Friend;E. Colleague;F. Work unit;G. Official or semi-official organizations such as Party, League, Trade Union, etc.;H. Non-official organizations such as religious and social organizations;

I. Other (please list)__________________

8. How do you confide when you are troubled? (Single option)

(l) Never tell anyone.

(2) Only one or twelve people with very close ties were reported.

(3) If a friend asks you, you will say it.

(4) Actively complain about your troubles in order to gain support and understanding.

9.How do you ask for help when you are in trouble? (Single option)

(l) Only rely on oneself, do not accept other people's help.

(2) Seldom ask for help from others.

(3) Sometimes ask for help from others.

(4)Often ask for help from my family, relatives and organizations, When in trouble.

10. For organizations (such as Party and League organizations, religious organizations, trade unions, student unions, etc.) to organize activities: (Single option)

(l) Never participate.

(2) Occasionally participate.

(3) Frequently participate.

(4) Actively participate.

Total score:

**Here are some questions about your experience with polycystic kidney disease**

1.How long did you diagnose polycystic kidney disease? ______ years

2. What was your last creatinine level?

________umol/L(______mg/ml) □Maintenance dialysis/Kidney transplantation

3. How did you find out that you had polycystic kidney disease?

□Family history of polycystic kidney disease, diagnosed by ultrasonography.

□Family history of polycystic kidney disease, diagnosed by genetic testing.

□Because kidney problems were found during a systematic examination.

□It's because other health problems are found during a systematic examination.

4. Does your father/mother know they have polycystic kidney disease?

□Know □Don't know □Uncertain

5. Do any of your family members have the following experiences? If so, please specify the number.

Dialysis,□Yes ______ people □ No

Kidney transplant,□Yes ______ people □ No

Subarachnoid hemorrhage,□Yes ______ people □ No

**Here are some questions about your willingness to have children**

6. Do you have any children?

□Yes ______ people □No(Jump to 8)

7. If the answer to your sixth question is “Yes”.

Sex of the first child ____ Age____,□The relevant examinations of polycystic kidney disease have been carried out, and the results are as follows:□Negative □Positive □Suspicious； □No relevant examinations were carried out.

Sex of the second child ____ Age____,□The relevant examinations of polycystic kidney disease have been carried out, and the results are as follows:□Negative □Positive □Suspicious； □No relevant examinations were carried out.

Sex of the third child ____ Age____,□The relevant examinations of polycystic kidney disease have been carried out, and the results are as follows:□Negative □Positive □Suspicious； □No relevant examinations were carried out.

8. Is your reproduction in line with the local family planning policy?

□Qualified □Non-conformity □Uncertain

9. Do you have any plans for reproduction?

□Yes □No □Uncertain

10. If you plan to have children in the future, are you worried that they will also have polycystic kidney disease?

□Worry □Don't worry □Uncertain

11. How strong is your desire to have children?

Not at all 0­­­­­—1­­­­­—2­­­­­—3­­­­­—4—5—6—7—8—9—10 Really want to

12. What are the main reasons why you don't want to have children?

□Family member structure is complete □Limited income □Concern about the heredity of polycystic kidney disease □Restricted by local fertility policy

□Physiological problems (including the elderly,combined with other diseases)

□Uncertain □Others, please specify _______________________

13. Do you want to interrupt the intensity of your desire to inherit the polycystic kidney disease gene to the next generation through existing technology?

Indifferent 0­­­­­—1­­­­­—2­­­­­—3­­­­­—4—5—6—7—8—9—10 Really want to

14. Have you ever had a gene test for polycystic kidney disease?

□Yes □No □Uncertain

**The next part is about your understanding and attitude to preimplantation genetic diagnosis (the third generation IVF technology).**

15. Before this questionnaire survey, did you know that genetic inheritance of polycystic kidney disease can be blocked by preimplantation genetic testing?

□Know □Hear nothing of（Jump to 17） □Uncertain（Jump to 17）

16. If the answer to your fifteenth question is "Know", how do you know it? (Multiple options)

□Direct introduction of medical workers □Direct introduction by non-medical workers □Television publicity □Network approach □Paper publicity materials □Others, please specify ______________

17. If you give birth, will you choose preimplantation genetic diagnosis?

□Yes □No（Jump to 20） □Uncertain（Jump to 20）

18. If the answer to your seventeenth question is yes, would you be willing to pay about 50,000 ¥ for preimplantation genetic diagnosis and in vitro baby?

□Willing □Unwilling □Uncertain

19. If the answer to your seventeenth question is “Yes”, will you choose prenatal diagnosis for further validation after preimplantation genetic diagnosis determines a normal pregnancy?

□Will choose（Jump to 21） □Would not □Uncertain

20. If the answer to your seventeenth question is "No" or "Uncertainty", what are your main concerns? (Multiple options)

□Technical safety issues □Technical Accuracy □Cost issues

□Complex and tedious procedures □It must be achieved by artificial insemination. □Low success rate

□Others, please specify ______________

21. What do you think about the safety of preimplantation genetic diagnosis?

□Have no idea □Very dangerous □Less dangerous □Fundamentally safe □Very safe

22. If you don't consider giving birth through preimplantation genetic testing, do you think this technology should be available to other patients with polycystic kidney disease?

□Should □Should not □Uncertain

**The next part is about your understanding and attitude towards prenatal diagnostic techniques.**

23. Before this questionnaire survey, did you know that prenatal diagnostic techniques can detect polycystic kidney disease gene mutations in the fetus?

□Know □Hear nothing of（Jump to 25） □Uncertain（Jump to25）

24. If your answer to question 23 is "Know", how do you know it? (Multiple options)

□Direct introduction of medical workers □Direct introduction by non-medical workers □Television publicity □Network approach □Paper publicity materials □Others, please specify ______________

25. If a couple or pregnant woman decides to terminate a pregnancy with normal fetal appearance, do you think this is acceptable?

□Acceptable □Unacceptable □Uncertain

26. If the fetus has serious genetic abnormalities, such as Down's syndrome, cystic fibrosis and so on, do you think termination of pregnancy is acceptable?

□Acceptable □Unacceptable □Uncertain

27. If the fetus is diagnosed as polycystic kidney disease by prenatal diagnosis, would you consider terminating pregnancy?

□Will consider □Would not □Uncertain

28. If you have a baby, would you choose prenatal diagnosis?

□Will choose（Jump to 30） □Would not □Uncertain

29. If your answer to question 28 is " Would not " or " Uncertain ", what are your main concerns? (Multiple options)

□Technical safety issues □Technical Accuracy □Cost issues

□Low success rate □Others, please specify ______________

**Costs for prenatal diagnosis and preimplantation genetic testing**

30. Do you have medical insurance? □Yes □No

31. The types of insurance are: □Medical Insurance for Urban Employees□Medical Insurance for Urban Residents

□New Rural Cooperative Medical Insurance □Commercial insurance □Cadre insurance □Major illness medical insurance □Others

32. Please estimate that the total cost of all kinds of medicines you pay for treatment in a year accounts for about ________% of your family's total income. The medical expenses paid by oneself last month are probably ____________

（Reference of medical expenses: Below 1000¥，1000-1999¥，2000-2999¥，3000-5000¥，Over 5000¥）

33. Considering that polycystic kidney disease can be passed on to the next generation, if prenatal diagnosis and preimplantation genetic testing technology can enter the medical insurance coverage, which of the following would you choose?

□I/We won't have any more children

□I/We'll have another baby, but we won't do any prenatal genetic testing.

□I/We'll have another baby and have prenatal diagnosis at the same time.

□I/We'll have another baby and preimplantation genetic diagnosis at the same time.

□If I / We want children, just consider adoption.

□I /We don't want any child.

34. If the total cost of pre-implantation genetic diagnosis and in vitro baby is about 50,000¥, how much do you think you can afford to pay for it personally?

□Within 1,000¥ □1,000-5000¥ □5,000-10,000¥

□10,000-20,000¥ □20,000-30,000¥ □More than 30,000¥

35. What is the approximate annual income of your family?

□Less than 10,000¥ □10,000-30,000¥ □30,000-50,000¥

□50,000-10,000¥ □More than 1,000¥

**Comments:** If so, please feel free to write down your comments on this questionnaire.

Thank you very much for your active cooperation in this questionnaire survey.

Your attitude is very helpful in formulating policies for preimplantation genetic diagnosis.
